# MiR-378a-5p Regulates Proliferation and Migration in Vascular Smooth Muscle Cell by Targeting CDK1

**DOI:** 10.3389/fgene.2019.00022

**Published:** 2019-02-19

**Authors:** Shaoyan Liu, Yanyan Yang, Shaoyan Jiang, Hong Xu, Ningning Tang, Amara Lobo, Rui Zhang, Song Liu, Tao Yu, Hui Xin

**Affiliations:** ^1^Department of Cardiology, The Affiliated Hospital of Qingdao University, Qingdao, China; ^2^Institute for Translational Medicine, Qingdao University, Qingdao, China; ^3^Department of Cardiology, The Affiliated Cardiovascular Hospital of Qingdao University, Qingdao, China; ^4^Department of Orthodontic, The Affiliated Hospital of Qingdao University, Qingdao, China

**Keywords:** miR-378a-5p, vascular smooth muscle cell, stent-restenosis, proliferation, migration, atherosclerosis, CDK1

## Abstract

**Objective:** Abnormal proliferation or migration of vascular smooth muscle cells (VSMCs) can lead to vessel lesions, resulting in atherosclerosis and in stent-restenosis (IRS). The purpose of our study was to establish the role of miR-378a-5p and its targets in regulating VSMCs function and IRS.

**Methods:** EdU assays and Cell Counting Kit-8 (CCK-8) assays were applied to evaluate VSMCs proliferation, wound healing assays and transwell assays were applied to assess cells migration. Furthermore, quantitative reverse transcription–polymerase chain reaction (qRT-PCR) was performed to investigate the expression level of miR-378a-5p IRS patients and healthy individuals. Target genes were predicted using Target Scan and miRanda software, and biological functions of candidate genes were explored through bioinformatics analysis. Moreover, RNA-binding protein immunoprecipitation (RIP) was carried out to analyze the miRNAs interactions with proteins. We also used Immunofluorescence (IF) and fluorescence microscopy to determine the binding properties, localization and expression of miR-378a-5p with downstream target CDK1.

**Results:** The expression of miR-378a-5p was increased in the group with stent restenosis compared with healthy people, as well as in the group which VSMCs stimulated by platelet-derived growth factor-BB (PDGF-BB) compared with NCs. MiR-378a-5p over-expression had significantly promoted proliferative and migratory effects, while miR-378a-5p inhibitor suppressed VSMC proliferation and migration. CDK1 was proved to be the functional target of miR-378a-5p in VSMCs. Encouragingly, the expression of miR-378a-5p was increased in patients with stent restenosis compared with healthy people, as well as in PDGF-BB-stimulated VSMCs compared with control cells. Furthermore, co-transfection experiments demonstrated that miR-378a-5p over-expression promoted proliferation and migration of VSMCs specifically by reducing CDK1 gene expression levels.

**Conclusion:** In this investigatory, we concluded that miR-378a-5p is a critical mediator in regulating VSMC proliferation and migration by targeting CDK1/p21 signaling pathway. Thereby, interventions aimed at miR-378a-5p may be of therapeutic application in the prevention and treatment of stent restenosis.

## Introduction

Coronary artery disease (CAD) is a serious disease threatening human health with its high mortality rate. Percutaneous Coronary Intervention (PCI) and stent implantation are commonly applied in the treatment of these obstructive diseases (Feinberg, 2014; Buccheri et al., 2016). However, there will be new atherosclerosis around stent implantation, and this will further increase the rate of restenosis after stent implantation (Wang H. et al., 2015; Liu et al., 2018). As a foreign substance, a scaffold can cause damage to the tunica intima, followed by inflammation and platelet aggregation in the damaged areas; resulting in the formation of plaque and thrombosis in the long term (Liu et al., 2018; Tang et al., 2018). And after stent implantation, endothelialization occurs gradually which cannot be removed from the blood vessels again; so, if in-stent restenosis occurs, stent implantation must be repeated (Alfonso et al., 2006; Finn et al., 2007). Aberrant proliferation and migration of Vascular smooth muscle cells (VSMCs) were identified as the main causes of these adverse events (Krist et al., 2015; Afzal et al., 2016; Huang et al., 2017). When VSMCs are stimulated, they promote the transfer of VSMCs from tunica media to tunica intima, from the contractile to the secretory, while stimulating free VSMCs and fibroblasts to secrete a large amount of extracellular matrix; the extracellular matrix is continuously deposited in the blood vessels, causing the intima to gradually thicken, resulting in stenosis (Braun-Dullaeus et al., 1998). Consequently, investigating key regulators and understanding the molecular mechanisms of VSMC biology has become a major method of treating atherosclerosis and stent restenosis.

MicroRNA (miRNA) is a type of small non-coding RNA that negatively modulates gene expression through mRNA translation repression or the induction of target mRNA instability (Gareri et al., 2016). Mounting shreds of evidence suggested that several miRNAs play critical roles in regulating VSMC proliferation and migration, such as miR-133, miR-221, miR-222, miR-663, miR-143, and miR-145 (Liu et al., 2009; Xin et al., 2009; Li et al., 2013; Mcdonald et al., 2015). Altering the expression of miRNA may have therapeutic potential in the prevention and treatment of stent restenosis.

It has been reported that the expression levels of miR-378a-5p in cardiac myocytes increased under some external stimuli (Wang et al., 2017; Rui et al., 2018). MiR-378a-5p is involved in the biological functions of some tumor cells (Luo et al., 2012), promoting proliferation in some tumor cells, and inhibiting proliferation in some others (Wang Z. et al., 2015). Considering that there may be tissue specificity associated with miR-378a-5p. However, the effect of miR-378a-5p in the regulation of VSMC biology remains unknown. The objective of the study is to investigate the potential roles of miR-378a-5p, as well as the molecular mechanisms of VSMCs proliferation and migration. Firstly, we screened and identified differential expression of miR-378 in patients with stent restenosis, then we studied the effect of up-regulation and down-regulation miR-378a-5p on the biological function of VSMCs. We also found that CDK1 was a potential gene target for the miR-378a-5p. Meanwhile, p21 may be the downstream target of CDK1. Consequently, miR-378a-5p is a key modulator to regulate proliferation and migration of VSMC partly by modulating the level of CDK1 gene expression. In this way, we have enough reason to believe that miR-378a-5p could be used as a diagnostic marker for early diagnosis, monitoring, and treatment of molecular targets for stent restenosis.

## Materials and Methods

### Blood Samples Acquisition and Baseline Clinical Characteristics Collection

Thirty-two persons were collected at the affiliated hospital of Qingdao University in Qingdao, China from June 2017 through February 2018. According to whether ISR was detected, they were classified into two groups: (1) The ISR group (*n* = 14): ISR is defined as a diameter stenosis greater than 50% in coronary angiography that occurs within the stent or 5 mm at the proximal or distal end of the stent; (2) The normal group (*n* = 18): 18 healthy persons without coronary heart disease as the control group. Basic information of all individuals collected, including age, gender, history of diabetes, drinking, hypertension, and smoking was noted. The research was supported by the Institutional Review Boards of Qingdao University Health Science Center. Paper version of informed consent was acquired from all subjects and the regional ethics committee in Qingdao, China approved the study protocol. The information of all clinical people is displayed in Supplementary Table 3.

### Test Animals

All experimental laboratory animals were approved by the Animal Care and Use Committee. C57BL/6 and ApoE-/- mice were purchased from Beijing Vital River Laboratory Animal Technology Co., Ltd. There were 3 mice in each group. The control group was given a normal diet, the experimental group was given a western diet (conventional mouse feed+0.15% cholesterol+21% fat) for 12 weeks, then cardiac blood was collected from mice weighing 25–30 g for further experiments.

### Cell Culture

The VSMC was purchased from the Chinese Type Culture Collection (Chinese Academy of Sciences, Shanghai, China) and cultured in Dulbecco’s modified Eagle’s medium (GIBCO, Grand Island, NY, United States) containing 10% fetal bovine serum (ExCell Bio.) in a 5% CO2 humidified incubator at 37°C. MiR-378a-5p mimics, miR-378a-5p inhibitor and negative control oligonucleotide (NC) (GenePharma, Shanghai, China) were transfected into the VSMCs using LipofectamineTM 2000 (Invitrogen, Grand Island, NY, United States).

### Western Blot Analysis

Cells lysates were prepared in buffer mixture containing 1 ml RIPA (Solarbio, Beijing, China), 0.1 mM PMSF reagent and a protease inhibitor cocktail (Roche, Basel, Switzerland) for 10 min on ice, after which protein samples were separated by 10% SDS–PAGE, then transferred into 0.45 μm polyvinylidene difluoride (PVDF) membrane, membranes were blocked with 5% not-fat milk in Tris-buffered saline-Tween 20 (TBS-T) for 1 h. And incubated with a rabbit anti-CDK1 monoclonal antibody (1:10000 dilution; Abcam, MA, United States) or anti-β-actin (1:2500 dilution; Cell Signaling Technology, United States). Then being washed three times with TBS-Tween 20, the secondary antibodies were added. Finally, the signals were visualized with Supersensitive ECL Chemiluminescent Kit, according to the directions of the manufacturer. The quantification of the protein bands was performed using ImageJ 1.8.0.

### RNA Extraction and qRT-PCR

Total RNA was extracted from the collected blood samples using TRIzol (Invitrogen, Grand Island, NY, United States), then treatment with DNase I (Takara, Otsu, Japan), then reverse RNA with reverse transcriptase kit (Takara) and mature miRNA levels were assessed using SYBR Green Real-time PCR Master Mix (Takara) according to the manufacturer’s guidance. The following primers which used in the experiment showed in Supplementary Tables 1, 2. U6 and GAPDH are based on different detection genes as reference genes, respectively. Analysis of qRT-PCR results using the 2 -ΔΔCt method.

### RNA Binding Protein Immunoprecipitation (RIP)

RNA-binding protein immunoprecipitation assays are performed to identify regions of the genome with RNA-binding proteins. In RIP assays, VSMCs were lysed in RIPA buffer containing 0.1 mM PMSF and 1% protease inhibitor cocktail on ice. After 10 min, the collecting cells were centrifuged at 12000 rpm for 20 min, the next step is to take 500 μg cell lysates incubated with the CDK1 antibody at 4°C overnight. Then add protein A/G-agarose beads and incubate for 4 h at 4°C with shaking. Immunoprecipitation separates RNA-binding proteins and their bound RNA. Furthermore, the combination of RIP and quantitative qRT-PCR can present experimental results more intuitively.

### Cell Proliferation

Cell Counting Kit-8 (CCK-8) assay was performed to assess VSMC proliferation. Cells were incubated in DMEM at a density of 5 × 10^3^ cells per well in 96-well plates for 24 h after transfection. And then the cells were maintained in 10 μl /well CCK-8 solution (7Sea-Cell Counting Kit, Shanghai, China) for an additional 1 h. Finally, the value was measured at 450 nm absorbance.

Another way to test cell proliferation is to use the EdU assay, VSMCs were cultured with EdU solution (50 nmol/L) (RiboBio, Guangzhou, China) for 2 h, then VSMCs were stained according to the product instructions. And finally, the pictures were obtained by a fluorescence microscope (Zeiss, LSM510, META). Image J 1.8.0 was used for analysis of the data.

### Cell Migration

Transwell assay and wound healing assay were performed to assess the migratory ability of VSMC.

Vascular smooth muscle cells were maintained in 6-well plates 12 h before transfection. After transfected, cells were cultured for 24 h in normal medium and then an additional 24 h in serum-free DMEM. After resuspending VSMCs in serum-free DMEM (5 × 10^5^ cells/ml), a mixture of 200 μl was added to the upper chamber of a transwell insert (Corning, Tewksbury, NY, United States) in a 24-well plate. Meanwhile, the lower chamber added 500 μl DMEM supplemented with 10% FBS. After 24 h incubation, PBS-rinsed cotton was used to wipe off the cells remaining on the upper side of the membrane. Then the cells were fixed with 4% paraformaldehyde for 1 h and dyed with 0.1% crystal violet for 30 min. After three washes, migrated VSMCs were recorded with a Zeiss LSM510 META microscope (20 × magnification), the 5 randomly fields were selected to count the cells.

#### Wound Healing Assay

Vascular smooth muscle cells were grown up to 60–70% in six-well plates. Then, cells were transfected with miR-378a-5p mimics, miR-378a-5p inhibitor, and NC. After 24 h, the wounds were made by a 1000-μl disposable pipette tip, which had reached almost 100% confluence. Distance on both sides of the scratch was visualized and photographed immediately and at different time points after wounding using a Zeiss LSM510 META microscope.

### Statistical Analysis

All data presented in this paper were the mean ± SD of at least three independent experiments, and an experiment performed with three samples for *in vitro* experiments. Data analyses were carried out using the GraphPad Prism 5 software. The quantitative data were presented as means ± SEM. Statistical analysis of the two groups by *t*-test, the different *P*-values indicate the different statistical significance: ^∗^*P* < 0.05, ^∗∗^*P* < 0.01, and ^∗∗∗^*P* < 0.001.

## Results

### MiR-378a-5p Expression in Stent-Restenosis Patients and ApoE -/- Mice

**FIGURE 1:**
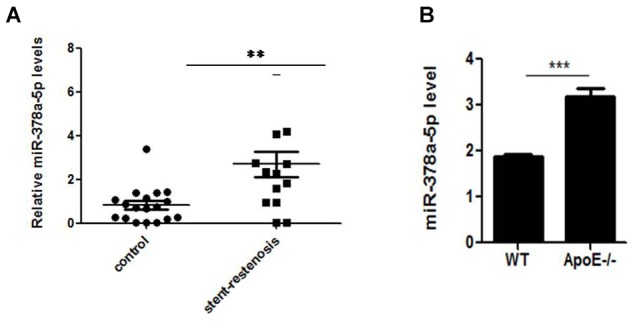
Independent validation of differential expression of miR-378a-5p. **(A)** Quantitative reverse transcription–polymerase chain reaction (qRT-PCR) for miR-378a-5p in an independent validation set of 14 stent-restenosis patients and 18 normal control subjects. The expression of miR-378a-5p in the two groups was normalized to U6 expression, ^∗∗^*p* < 0.01. **(B)** MiR-378a-5p transcript expression in atherosclerotic plaques of ApoE knockout (ApoE-/-) mice and wild-type (WT) C57 control mice was measured by qRT-PCR, ^∗∗∗^*p* < 0.001.

MiR-378a-5p expression were detected between the patients with stent-restenosis and control group, respectively, by qRT-PCR, in which we found that miR-378a-5p expression levels were upregulated in stent-restenosis patients compared with control group (Figure 1A); MiR-378a-5p expression levels were higher in atherosclerotic plaques of ApoE knockout (ApoE -/-) mice than in control subjects as measured by qRT-PCR (Figure 1B).

### MiR-378a-5p Promoted VSMCs Proliferation and Migration

**FIGURE 2:**
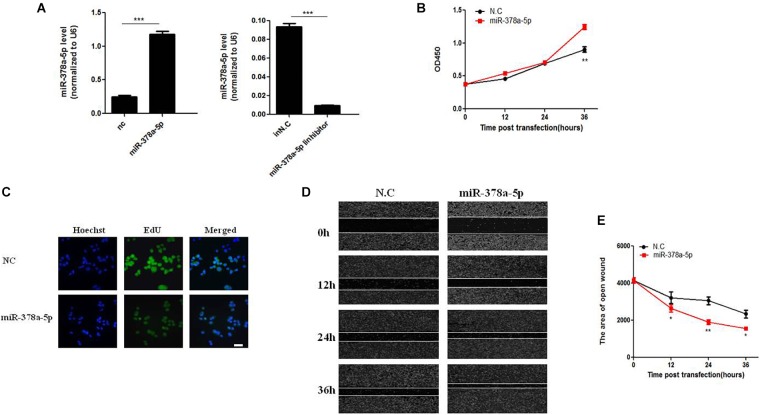
Effects of miR-378a-5p on the proliferation and migration in vascular smooth muscle cell (VSMC). **(A)** Expression levels of miR-378a-5p were examined by qRT-PCR after transfection of miR-378a-5p mimics, miR-378a-5p inhibitor and negative control oligonucleotide (NC), ^∗∗∗^*p* < 0.001. **(B)** The CCK-8 assay was performed to investigate the proliferation of VSMCs with miR-378a-5p mimics, miR-378a-5p inhibitor transfection, at 0, 12, 24, and 36 h, respectively. **(C)** Representative micrographs of EdU staining of VSMCs, with control or miR-378a-5p transfection, scale bar = 20 μm. **(D,E)** miR-378a-5p effects on VSMC migration ability as determined via classic scratch assay and its quantification analysis, all images were taken under the same magnification, data are presented as mean ± SEM, ^∗^*p* < 0.05 and ^∗∗^*p* < 0.01.

MiR-378a-5p regulates biological function of VSMC. VSMCs were transfected with miR-378a-5p mimics (25 nM) and miR-378a-5p inhibitor (100 nM), both of them had visible transfection efficiency (Figure 2A). MiR-378a-5p mimics-transfected cells have stronger proliferative capacity compared with NC (25 nmol/L) (Figure 2B). Meanwhile, VSMC proliferation was assessed using the EdU assay; representative staining of the nucleus of proliferating VSMCs was shown in Figure 2C. EdU incorporation measured by confocal laser microscopy. In addition, we observed that miR-378a-5p significantly promoted cell migration in miR-378a-5p mimics-transfected cells compared with NC, the miR-378a-5p inhibitor has the opposite effect (Figure 2D,E). Sequence of RNAs used in this study showed in Supplementary Table 4.

### MiR-378a-5p Promoted Proliferation and Migration in PDGF-BB-Stimulated VSMCs

**FIGURE 3:**
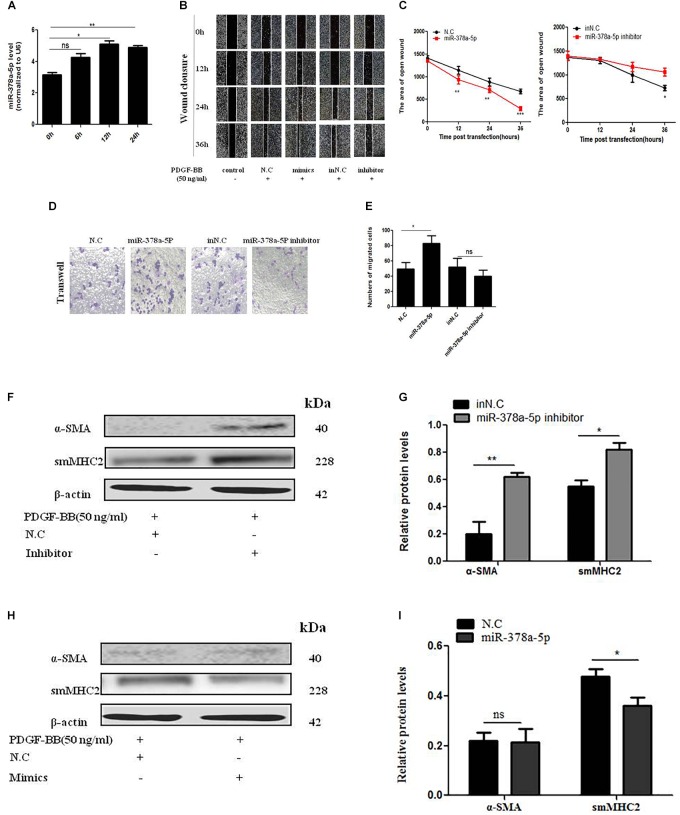
MiR-378a-5p promotes platelet-derived growth factor-BB (PDGF-BB)-induced VSMC proliferation and migration. **(A)** miR-378a-5p expression levels were increased in VSMCs under the time gradient of PDGF-BB-induced (50 ng/ml), compared with quiescent cells, as demonstrated using qRT-PCR, data are presented as mean ± SEM. ^∗^*p* < 0.05 and ^∗∗^*p* < 0.01. **(B,C)** miR-378a-5p abrogated PDGF-BB-mediated effects on VSMC migration ability as determined via classic scratch assay and its quantification analysis, all images were taken under the same magnification. **(D,E)** The miR-378a-5p mimics significantly increased PDGF-BB-induced (50 ng/ml) VSMC migration, as determined by transwell assay (original magnification: × 200) and its quantification analysis, data are presented as mean ± SEM. ^∗^*p* < 0.05. **(F)** Representative western blots of VSMC phenotype marker genes transfected with miR-378a-5p inhibitor compared with in NC under the condition with PDGF-BB (50 ng/ml) stimulated for 12 h. **(G)** Quantitative analysis of differentiation marker gene expression in the two groups, data are presented as mean ± SEM. ^∗^*p* < 0.05 and ^∗∗^*p* < 0.01. **(H)** Representative western blots of VSMC phenotype marker genes transfected with miR-378a-5p mimics compared with NC under the condition with PDGF-BB (50 ng/ml) stimulated for 12 h. **(I)** Quantitative analysis of differentiation marker gene expression in the two groups, data are presented as mean ± SEM. ^∗^*p* < 0.05.

In order to detect the effects of miR-378a-5p in PDGF-BB-stimulated VSMC, we transfect miR-378a-5p mimics, miR-378a-5p inhibitor and NC into VSMCs. MiR-378a-5p was upregulated after PDGF-BB treatment in a time-dependent manner (Figure 3A). MiR-378a-5p mimic-induce miR-378a-5p up-regulation significantly increased VSMC migration compared with NC, the miR-378a-5p inhibitors have the opposite effect, as demonstrated by wound closure assay (Figure 3B,C) and transwell assay (Figure 3D,E). To detect the effect of PDGF-BB on VSMC phenotype switching genes, VSMCs were treated with PDGF-BB (50 ng/ml) for 12 h, the expression levels of sm-MHC2 and α-SMA protein decreased after being transfected with miR-378a-5p mimics (Figure 3F–I). These results demonstrate that miR-378a-5p promoted proliferation and migration in PDGF-BB-stimulated VSMCs.

### Identification of CDK1 as a Direct Target of MiR-378a-5p in VSMCs

**FIGURE 4:**
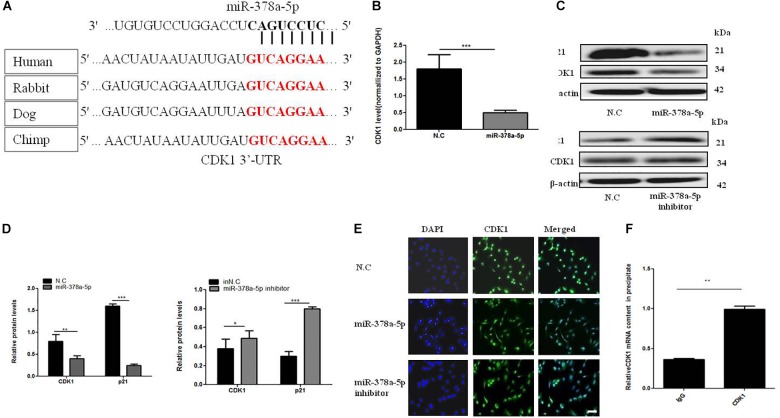
MiR-378a-5p participates in the regulation of *CDK1* expression. **(A)** There is a potential seed sequence of miR-378a-5p in the 3′UTR of CDK1, the putative seed sequences for the 3′-UTR of CDK1 were highly conserved in different species. **(B)** qRT-PCR, **(C,D)** western blot were used to determine CDK1 mRNA and protein expression levels and p21 protein levels in VSMCs transfected with miR-378a-5p mimics (25 nmol/L), inhibitor (100 nmol/L), NC (25 nmol/L) and inNC (25 nmol/L) after PDGF-BB stimulation, data are presented as mean ± SEM, ^∗^*p* < 0.05, ^∗∗^*p* < 0.01, and ^∗∗∗^*p* < 0.001. **(E)** Typical fluorescence photomicrograph by laser scan confocal microscopy, scale bar = 20 μm. The photographs of nuclei (blue) and CDK1 (green) fluorescence were taken under a same field and then were merged. **(F)** Real-time PCR validation of CDK1-associated mRNAs identified by RIP, data are presented as mean ± SEM. ^∗∗^*p* < 0.01.

Target Scan algorithms found that CDK1 was a potential miR-378a-5p target. We found that the putative seed sequences for miR-378a-5p within the 3′-UTR of CDK1 were highly conserved and there has a potential seed sequence of miR-378a-5p in the 3′UTR of CDK1 (Figure 4A). To illustrate the relationship between CDK1 and miR-378a-5p, we transfected VSMCs with miR-378a-5p mimics, inhibitor and NC, and investigated CDK1 expression using western blot analysis and qRT-PCR, overexpression of miR-378a-5p suppressed the protein expression of CDK1, as showed in Figure 4B–D, referring to the literature, p21 is one of the downstream targets of CDK1 (Kreis et al., 2016). In our experiment, we found that miR-378a-5p works by reducing CDK1 and then regulating p21. Then we performed immunofluorescence which showed that CDK1 protein expression was decreased with transfected miR-378a-5p mimics compared with NC, as showed in Figure 4E. The conclusion of these findings is: miR-378a-5p upregulation could inhibit CDK1 expression at the post-transcriptional level. RIP was used to analyze the protein interactions with CDK1 mRNA. The % input detected for CDK1 immunoprecipitation is above that detected for IgG immunoprecipitation, which means CDK1 antibody could pull down more miR-378a-5p than the non-specific IgG antibody (Figure 4F). These results demonstrated that miR-378a-5p directly binds to the 3′-UTR of CDK1.

### CDK1 Is Involved in VSMC Proliferation and Migration

**FIGURE 5:**
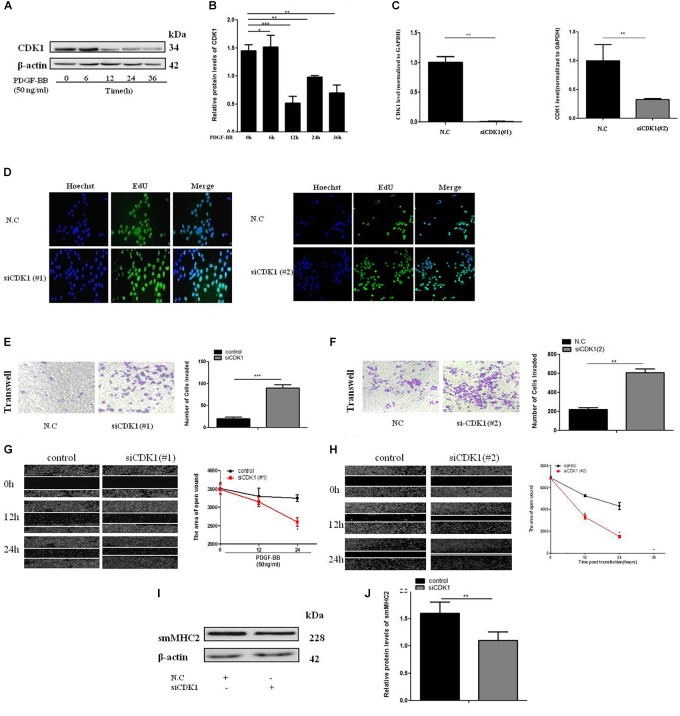
CDK1 regulates VSMC proliferation and migration. **(A)** PDGF-BB (50 ng/ml) caused a time-dependent decrease in CDK1 expression levels as determined by western blot. **(B)** Quantitative western blot analysis of CDK1 expression in PDGF-BB-stimulated (50 ng/ml) VSMCs at various time points by western blot, data are presented as mean ± SEM. ^∗^*p* < 0.05, ^∗∗^*p* < 0.01, and ^∗∗∗^*p* < 0.001. **(C)** The expression level of CDK1 was detected by qRT-PCR, after transfection with siCDK1 in VSMCs, ^∗^*p* < 0.05. **(D)** siCDK1 increased the proliferation of VSMCs, as determined by EdU assays, scale bar = 20 μm. **(E,F)** Transwell were used to detect of VSMCs proliferation and migration, (Original magnification: × 200), ^∗∗∗^*p* < 0.001. **(G,H)** The wound closure assay was performed to investigate the migration ability of VSMCs under PDGF-BB-induced (50 ng/ml). **(I)** Representative western blots of VSMC phenotype marker genes transfected siCDK1 compared with control. **(J)** Quantitative analysis of differentiation marker gene expression in the two groups, data are presented as mean ± SEM, ^∗∗∗^*p* < 0.001.

To determine the effect of CDK1 on the VSMC, expression of CDK1 under PDGF-BB stimulation gradient by western blot (Figure 5A,B). Two SiRNAs were designed to knock down CDK1, the sequences were shown in Supplementary Table 2. The transfection efficiency and expression efficiency of siCDK1 was detected by qRT-PCR. The result showed that CDK1 was significantly down-regulated when transfected with siCDK1(#1) and siCDK1(#2), respectively (Figure 5C). The influence of siCDK1 on the proliferation of cells was assessed by EdU assay (Figure 5D). Transwell assay was applied to investigate the migration ability of cells, showed in Figure 5E,F. The wound closure assay was performed to detect the migration ability of cells with or without PDGF-BB-induced (50 ng/ml) (Figure 5G,H). α-SMA and sm-MHC2 protein level were down-regulated after transfected with siCDK1 (Figure 5I,J). The findings showed that the proliferation and migration activity of VSMCs transfected with siCDK1 was higher than that of cells transfected with an empty plasmid with or without PDGF-BB stimulation.

### MiR-378a-5p Targeted CDK1 Expression and Enhanced Migration of VSMCs

**FIGURE 6:**
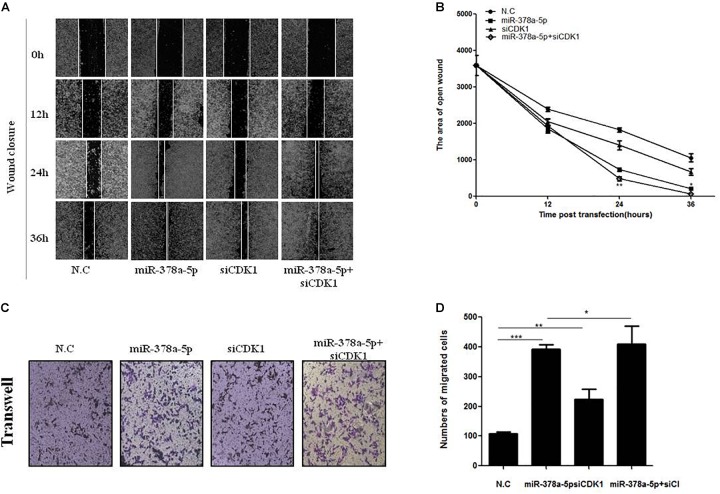
CDK1 was involved in miR-378a-5p-mediated cellular effects. **(A,B)** Low expression of CDK1 promoted VSMC migration and significantly promoted the migratory effects of miR-378a-5p on VSMCs, as determined by wound closure and its quantification analysis, data are presented as mean ± SEM. ^∗^*p* < 0.05 and ^∗∗^*p* < 0.01. **(C,D)** The same effects was also determined by transwell assay (original magnificaton: × 200) and its quantification analysis, data are presented as mean ± SEM. ^∗^*p* < 0.05, ^∗∗^*p* < 0.01, and ^∗∗∗^*p* < 0.001.

For further confirm whether CDK1 is a functional target gene of miR-378a-5p, wound closure (Figure 6A,B) and transwell assays (Figure 6C,D) were used to measure the migratory ability of VSMCs, compared with the NC, the migration rate of the miR-378a-5p mimic group was remarkably increased, while that of the si-CDK1 group has no significant increase, the group with miR-378a-5p mimic + siCDK1 restored the migration ability, the number of cells migrated significantly increased. From these results, we determined that CDK1 is a functional downstream target of miR-378a-5p.

## Conclusion

In our study, we identified that miR-378a-5p is an important modulator in the PDGF-BB stimulated proliferation and migration of VSMC by targeting, at least partly CDK1 pathway. Also, miR-378a-5p acts on CDK1 and then partly affects p21 to play a role in cell function. In addition, miR-378a-5p negatively regulates the expression of CDK1 after PDGF-BB serve as a stimulant to promote VSMC proliferation. SiCDK1 can partially recover the proliferation of VSMC by PDGF-BB. Furthermore, miR-378a-5p expression levels were upregulated in both human atherosclerotic vascular tissues and proliferation VSMC. The conclusion of these results is: miR-378a-5p/CDK1/p21 is a considerable pathway that can be used as a new therapeutic target in the prevention of atherosclerosis and stent restenosis.

## Discussion

According to the National Health and Family Planning Commission, in the year 2015 more than 500,000 patients with coronary heart disease in mainland China need PCI. PCI has become the main revascularization strategy for unstable coronary artery disease. Despite this, PCI itself still has a problem that has not been overcome, ISR. DES placement does reduce the incidence of ISR, but its incidence is still as high as 10% (Pleva et al., 2018). ISR greatly limits the benefits of PCI. The prevention of ISR is still an important concern. A study found that the ISR of DES is mainly the result of the proliferation of VSMCs, and the high-pressure effect of post-stent expansion accelerates the proliferation of VSMCs. Proliferation, migration, and formation of the extracellular matrix of VSMCS in the middle of the blood vessels lead to intimal regeneration and stenosis of the lumen (Ko et al., 2012). Stent implantation must be performed again for patients with restenosis (Miziastec et al., 2009), so the targeted regulation of VSMC is of great significance for the treatment and prevention of post-stent restenosis.

Accumulating reports have been made to understand the effect of miRNAs in VSMCs biology (Choe et al., 2013), but the specific molecular mechanism is still unknown. In our study, we demonstrated that targeting of the miR-378a-5p/CDK1/p21 pathway may be a potential therapeutic method for stent restenosis.

MiR-378a is a small non-coding RNA molecule which has two mature chains: (1) miR-378a-3p, (2) miR-378a-5p (Krist et al., 2015). The early study of the miR-378a-5p is mainly based on its relationship with the occurrence and development of tumors. However, the role of miR-378a-5p in the regulation of VSMCs requires deeper research. To explore the effects of miR-378a-5p in VSMCs in restenosis, we performed CCK-8 and EdU assays to detect VSMC proliferation, wound healing and transwell assays to evaluate VSMC migration. In the end, we come to this conclusion that miR-378a-5p plays a role in regulating the proliferation, migration and phenotypic transformation of VSMCs, that is miR-378a-5p participates in the abnormal VSMC biology functions that contribute to stent restenosis development.

Cyclin-dependent kinase 1 (CDK1) is a protein that regulates the cell cycle, which belongs to a serine/threonine kinase family (Malumbres and Barbacid, 2009); previous studies have proved that CDK1 acted as a key regulator for cell cycle (Yang et al., 2016), and its expression increases in several cancer growths, such as colon carcinoma (Meyer et al., 2009), non-small cell lung cancer tumor (Kim et al., 2008; Zhang et al., 2011); There are also researches that identify that inhibition of CDK1 can suppress the proliferation and migration of some tumor cells (Zhang et al., 2015). Interestingly, in oral squamous cell carcinoma (OSCC), the expression of CDK1 increases with the progression of tumor stage, but the expression of CDK1 is reduced at the stage IV and late stage of the tumor (Chen et al., 2015). There is also a report that suggests overexpression of CDK1 inhibits cell proliferation. CDK1 expression increased or decreased at different time intervals (Rui et al., 2011), this phenomenon is hard to explain from a biological view. So maybe miRNA plays different roles in regulating CDK1 at different time points; the effect of CDK1 on cell proliferation cycle needs to be further studied.

Although our research has demonstrated that miR-378a- 5p can target CDK1 to regulate proliferation and migration of VSMCs, but (1) there have been studies that show one miRNA could regulate many target genes, meanwhile, one gene could be modulated by different miRNA. Therefore, targeting miR-378a-5p to treat atherosclerosis and stent restenosis may also affect other genes related to the proliferation of VSMCs, establish an interactive network of non-coding RNAs related to restenosis for early prediction and prognostic evaluation purposes. (2) Non-coding RNA also has problems in application technology and security. (3) Non-coding RNAs also present application technique and safety issues, such as how to coat miR-378a-5p onto a scaffold, and to understand the effect of the release on human body. (4) Chemically synthesized miRNA inhibitor and mimics are used as scaffold coatings, considering factors such as *in vivo* concentration, half-life, dose, and sample specificity. (5) Another important consideration is whether external miRNAs will affect normal genes. In this research, there is still a lack of experiments on the downstream target p21 of CDK1, which needs further verification. Moreover, the selection of samples has some limitations, the number of samples is small, so the clinical sample size should be increased. The specific mechanism of miR-378a-5p for target regulation of atherosclerosis and stent restenosis remains to be further studied in the follow-up work.

## Author Contributions

SL and NT carried out the cell and protein analysis. YY carried out the molecular experiments. SJ, HX, RZ, and SL performed the clinical analysis. TY and SL participated in the data analysis, performed the statistical analysis, and drafted the manuscript. TY and HX conceived and designed the study, participated in the data analysis and coordination, and helped to draft the manuscript. All authors read and approved the final manuscript.

## Conflict of Interest Statement

The authors declare that the research was conducted in the absence of any commercial or financial relationships that could be construed as a potential conflict of interest.
