# Corrigendum: MiR-378a-5p Regulates Proliferation and Migration in Vascular Smooth Muscle Cell by Targeting CDK1

**DOI:** 10.3389/fgene.2019.00193

**Published:** 2019-03-15

**Authors:** Shaoyan Liu, Yanyan Yang, Shaoyan Jiang, Hong Xu, Ningning Tang, Amara Lobo, Rui Zhang, Song Liu, Tao Yu, Hui Xin

**Affiliations:** ^1^Department of Cardiology, The Affiliated Hospital of Qingdao University, Qingdao, China; ^2^Institute for Translational Medicine, Qingdao University, Qingdao, China; ^3^Department of Cardiology, The Affiliated Cardiovascular Hospital of Qingdao University, Qingdao, China; ^4^Department of Orthodontic, The Affiliated Hospital of Qingdao University, Qingdao, China

**Keywords:** miR-378a-5p, vascular smooth muscle cell, stent-restenosis, proliferation, migration, atherosclerosis, CDK1

In the original article, we neglected to include the funder “National Natural Science Foundation of China,” “81870331” to TY.

Additionally, there was an error in affiliation 3. Instead of “Department of Cardiology, The Affiliated Hospital of Qingdao University, Qingdao, China,” it should be “Department of Cardiology, The Affiliated Cardiovascular Hospital of Qingdao University, Qingdao, China.”

The authors apologize for these errors and state that this does not change the scientific conclusions of the article in any way. The original article has been updated.

