# Supplementary material for: MiR-378a-5p Regulates Proliferation and Migration in Vascular Smooth Muscle Cell by Targeting CDK1
Source: Front Genet. 2019 Feb 19;10:22. doi: 10.3389/fgene.2019.00022 (PMC6389607; doi:10.3389/fgene.2019.00022)
Supplement: Supplementary file 1 [file Table_1.DOCX]

**Table 1. Sequence of RNAs used in this study**

**Name**  **Sequence**

mimics N.C UUCUCCGAACGUGUCACGUTT

miR-378a-5p mimics CUCCUGACUCCAGGUCCUGUGU

inhibitor N.C CAGUACUUUUGUGUAGUACAA

miR-378a-5pinhibitor ACACAGGACCUGGAGUCAGGAG
