# Supplementary material for: MiR-378a-5p Regulates Proliferation and Migration in Vascular Smooth Muscle Cell by Targeting CDK1
Source: Front Genet. 2019 Feb 19;10:22. doi: 10.3389/fgene.2019.00022 (PMC6389607; doi:10.3389/fgene.2019.00022)
Supplement: Supplementary file 2 [file Table_2.docx]

**Table 2. The si-RNA sequences for knock-down of CDK1 expression in this study**

**Name Sequence**

siCDK1(#1)sense strand 5′-GGCACUGAAUCAUCCAUAUTT-3′

siCDK1(#1) antisense strand 5′-AUAUGGAUGAUUCAGUGCCTT-3′

siCDK1(#2)sense strand 5′-CCUGGUCAGUACAUGGAUUdTdT-3′

siCDK1(#2) antisense strand 5′-AAUCCAUGUAC UGACCAGGdTdT-3′
