# Supplementary material for: MiR-378a-5p Regulates Proliferation and Migration in Vascular Smooth Muscle Cell by Targeting CDK1
Source: Front Genet. 2019 Feb 19;10:22. doi: 10.3389/fgene.2019.00022 (PMC6389607; doi:10.3389/fgene.2019.00022)
Supplement: Supplementary file 3 [file Table_3.DOCX]

**Table 3. Sequence of the PCR primers used in this study**

**Name Sequence (5’-3’)**

miR-378a-5p-Forward CTCCTGACTCCAGGTCCTGT

CDK1-Forward CATGGATTCTTCACTTGTTAAGGT

CDK1-Reverse TCCACTTCTGGCCACACTTC

GAPDH-Forward CTGACTTCAACAGCGACACC

GAPDH-Reverse TGCTGTAGCCAAATTCGTTGT
