# Supplementary material for: MiR-378a-5p Regulates Proliferation and Migration in Vascular Smooth Muscle Cell by Targeting CDK1
Source: Front Genet. 2019 Feb 19;10:22. doi: 10.3389/fgene.2019.00022 (PMC6389607; doi:10.3389/fgene.2019.00022)
Supplement: Supplementary file 4 [file Table_4.DOCX]

**Table 4．Patients and donors Information**

| **NO.** | **Gender** | **Age** | **Diagnosis** | **Smoking** | **Alcohol** | **Hypertension** | **T2MD** |
| --- | --- | --- | --- | --- | --- | --- | --- |
| 1 | F | 67 | stent-restenosis | - | - | + | - |
| 2 | M | 42 | stent-restenosis | + | + | + | - |
| 3 | M | 60 | stent-restenosis | + | + | + | - |
| 4 | M | 58 | stent-restenosis | - | + | - | - |
| 5 | M | 42 | stent-restenosis | + | + | + | - |
| 6 | M | 69 | stent-restenosis | + | + | + | + |
| 7 | M | 63 | stent-restenosis | - | - | - | + |
| 8 | M | 51 | stent-restenosis | + | + | - | - |
| 9 | F | 64 | stent-restenosis | - | - | + | + |
| 10 | M | 63 | stent-restenosis | - | - | + | - |
| 11 | M | 79 | stent-restenosis | + | + | + | - |
| 12 | M | 45 | stent-restenosis | - | - | - | - |
| 13 | F | 70 | stent-restenosis | - | - | + | + |
| 14 | F | 59 | stent-restenosis | - | + | - | + |
| 15 | M | 28 | control | + | + | - | - |
| 16 | M | 32 | control | + | + | - | - |
| 17 | M | 32 | control | - | - | - | - |
| 18 | M | 44 | control | - | + | - | - |
| 19 | M | 47 | control | + | + | + | - |
| 20 | M | 36 | control | - | + | - | - |
| 21 | M | 32 | control | - | - | - | - |
| 22 | F | 35 | control | - | - | - | - |
| 23 | F | 25 | control | - | - | - | - |
| 24 | F | 28 | control | - | - | - | - |
| 25 | F | 28 | control | - | - | - | - |
| 26 | F | 45 | control | - | + | - | - |
| 27 | F | 45 | control | - | + | + | - |
| 28 | F | 43 | control | - | - | - | - |
| 29 | F | 43 | control | - | - | - | - |
| 30 | F | 36 | control | - | - | - | - |
| 31 | F | 38 | control | - | - | - | - |
| 32 | F | 41 | control | - | - | - | - |
